# Exploring public support for novel tobacco and alcohol control policies in Great Britain 2021–2023: A population-based cross-sectional survey

**DOI:** 10.1016/j.heliyon.2024.e41303

**Published:** 2024-12-16

**Authors:** Vera Helen Buss, Lion Shahab, Sharon Cox, Loren Kock, Melissa Oldham, Linda Bauld, Hazel Cheeseman, Garth Reid, Jamie Brown

**Affiliations:** a Department of Behavioural Science and Health, University College London, UK; b Spectrum Research Consortium, UK; c Behavioural Research UK, UK; d Vermont Center on Behavior and Health, University of Vermont, Vermont, USA; e Usher Institute, University of Edinburgh, UK; f Action on Smoking and Health (ASH), London, UK; g Public Health Scotland, Edinburgh, UK

**Keywords:** Tobacco control, Alcohol control, Public opinion, Inequalities, Socio-economic status

## Abstract

**Objective and rationale:**

This study assessed support for novel tobacco compared with alcohol control policies among adults in Great Britain in 2021–2023. Objectives were to assess 1) overall level of support for tobacco compared to alcohol control policies; 2) level of support for tobacco compared to alcohol control policies among people who smoke tobacco or who consume alcohol at increasing and higher risk levels, or who do both; 3) level of support for tobacco compared to alcohol control policies among different sociodemographic groups?

**Methods:**

Data were collected in September/October 2021–2023 in a monthly population-based survey on smoking and drinking behaviour of adults across Great Britain (N = 6311), weighted to match the overall population. Outcome measure was level of support for each seven tobacco and alcohol control policies.

**Results:**

More people were in support of tobacco than alcohol control policies (e.g., 57 % vs. 51 % for tax-related policies). This trend was apparent across all sociodemographic subgroups. With one exception, the policies included in this study were supported by more than half of the participants. The exception was decreasing the visibility of alcohol products in shops, which received 41 % of support. People who engaged in the behaviour targeted by policies (tobacco more so than alcohol use) were generally less supportive.

**Conclusion:**

Overall, public support for tobacco and alcohol control policies is high in Great Britain. With one exception, the policies were supported by over half of participants, suggesting that the public is in favour of government regulations to reduce smoking and drinking prevalence in Great Britain.

## Highlights

• People who are directly affected by a policy were less likely to support it.
• People were highly supportive of policies offering support to individuals.
• There was also high support for the policy that is aimed at protecting youth.
• People were less supportive of more intrusive policies.
• The results indicated that a policy gets higher support after being implemented.

## 1 Introduction

Tobacco smoking and alcohol consumption are both substantial contributors to morbidity and mortality worldwide [1]. In Great Britain, smoking rates have been in decline for a long time, driven by a range of tobacco control measures [2,3]. However, since the start of the COVID pandemic, declines in smoking prevalence have stagnated [4]. Further tobacco control measures are likely required to reach the UK Government's goal of a smokefree nation by 2030 (i.e., smoking prevalence below 5 %) [5]. For alcohol consumption, rates of harmful levels have fluctuated over the past decades in Great Britain [[6], [7], [8]]. During the COVID pandemic, prevalence of increasing and higher risk drinking significantly increased [9,10]. Despite its substantial public health impact [11], the UK Government has not issued an alcohol control strategy since 2012 [12]. The present study aimed to assess the extent to which the public in Great Britain supports novel tobacco compared to similar alcohol control measures.

Policymakers consider the public's opinion on new health policies when deciding on whether they should be implemented [13,14]. Comparing tobacco to alcohol control policies, Western societies and their parliamentary representatives tend to show higher support for the former than for the latter [15,16]. This difference is currently reflected in stricter regulations for tobacco than for alcohol [17]. The present study aimed to understand whether this general trend differs by the type of policy (i.e., through which mechanisms the policy aims to regulate behaviour), by whether people engage in the behaviour targeted by the policy (i.e., smoking tobacco or drinking alcohol at increasing and higher risk levels), and by different sociodemographic characteristics (i.e., gender, age, socioeconomic position, and level of education) and in which part of Great Britain they live (i.e., England, Scotland, or Wales).

According to a framework developed by Grelle and Hofman [18], problem awareness and the desire for governmental support on the intended target of a policy both contribute to whether a person supports a policy. The two factors are interconnected and support-seeking characteristics of the individual influence this association. These characteristics are personal and political attitudes and beliefs, for example, the degree to which people trust the government to proceed in accordance with their interests and values in the context of the tobacco or alcohol control. The support-seeking characteristics are in turn shaped by factors such as the age, gender and socioeconomic position [18]. In a systematic review, Diepeveen et al. [15] found that women and older people were more likely to support health policies than men and younger people. There was no clear association with socioeconomic position [15]. Results for differences in policy support by educational level are mixed. For tobacco control policies, Doucet et al. [19] reported that people with a higher educational attainment expressed a higher level of support for policies. For alcohol control policies, the direction of the association differed by the type of policy [20,21].

Kock et al. [22] reported broadly similar levels of support for novel tobacco control policies between England, Scotland, and Wales. Comparable data for alcohol control policies have not been published. However, there are noteworthy differences in the prevalence of increasing and higher risk drinking and the policies that have already been implemented across the three nations. For example, Scotland has higher rates of increasing and higher risk drinking than the other two nations [23], but also stricter legislation [21]. There is an association between policy acceptance and compliance [18]. A deeper understanding about who supports different policies can therefore provide insights into who is likely to comply with them if they are implemented. However, the relationship between support prior to policy implementation and compliance is complex [18]. For example, support by a sufficiently large minority group (‘critical mass’) can create social-change dynamics, ultimately leading to compliance by a large part of the population [18,24]. On the other hand, people may state that they support a policy but still not comply with it (attitudes vs. actions) [18,[25], [26], [27]].

This study aims to build a better understanding of the behavioural and sociodemographic correlates of support for tobacco and alcohol control policies, potentially revealing strategies to boost government confidence in implementing alcohol regulation despite current reluctance. It may also offer broader insights into why the public supports certain policies over others. The study descriptively explores differences in public support for various policies with potential to be implemented in the UK, focussing on aspects related to the target behaviour (tobacco smoking or alcohol consumption), policy category, target population (e.g., young people, people with alcohol dependence), and sociodemographic characteristics (gender, age, socioeconomic position, education level, nation within Great Britain). The study data were collected at a time of new policy announcements and speculation around potential new measures being implemented, providing a rare snapshot into immediate responses to such changes in public discourse. This study assessed the following research questions asked of adults in Great Britain between 2021 and 2023: (1) What is the overall level of support for tobacco compared to alcohol control policies? (2) What is the level of support for tobacco compared to alcohol control policies among people who smoke tobacco or who consume alcohol at increasing and higher risk levels, or who do both? (3) What is the level of support for tobacco compared to alcohol control policies among different sociodemographic groups, including differences between nations?

## 2 Methods

### 2.1 Study design and participants

The study used data collected as part of the Smoking and Alcohol Toolkit Study, a population-based repeat cross-sectional study in Great Britain [28]. Every month, approximately 2450 households are selected using a hybrid approach involving random location and quota sampling to balance out expected non-response effects within the randomly sampled output areas. Randomly chosen locations are derived from 227,403 output areas, stratified based on an established geo-demographic classification of the British population, with each area comprising roughly 300 households. A market company collects data via phone interviews with one household member until monthly quotas are met and shares the anonymised data with the research team. The survey, which encompasses questions on sociodemographic characteristics, smoking and alcohol measures, incorporated policy support questions in one month of each of the last three years (September 2021, October 2022, October 2023). The study was pre-registered, including the analysis plan, on the Open Science Framework (https://osf.io/cg2x5/), and the manuscript adheres to the Strengthening the Reporting of Observational Studies in Epidemiology (STROBE) statement [29]. We discussed the results of the analysis with the University College London Tobacco and Alcohol Research Public and Patient Involvement (PPI) group, which consists of people with experience of tobacco and alcohol use, to explore potential explanations for the results.

### 2.2 Outcome variables and covariates

The outcome measure was level of support for different tobacco and alcohol control policies (Table 1). Participants were asked: “To what extent do you support or oppose this policy suggestion?“, with responses recorded as (i) ‘Strongly support’, (ii) ‘Tend to support’, (iii) ‘No opinion either way’, (iv) ‘Tend to oppose’, (v) ‘Strongly oppose’ or (vi) ‘Unsure/Don't know’. The policy suggestions were read in randomised order, with tobacco and alcohol policies asked separately and not directly after another. For the first research question, level of support was categorised as ‘supporting’ (response options i and ii), ‘opposing’ (response options iv and v), or ‘indecisive’ (response options iii and vi). For the second and third research question, responses of ‘opposing’ and ‘indecisive’ were combined to create a binary outcome variable (‘supportive’ vs. ‘not supportive’). To enable easier comparisons between tobacco and alcohol control policies, we grouped them into one of five categories (Table 1). All these policies have been proposed for implementation in the UK in recent years. As tobacco products are more strictly regulated than alcohol products and the measures currently being considered for the products differ, not all policies in Table 1 are directly comparable for tobacco and alcohol.

**Table 1:** Policy categories and respective tobacco and alcohol control policy statements included in the study.

Category	Tobacco Control Policy	Alcohol Control Policy
Tax	“Increasing tax on cigarettes and tobacco substantially above the annual rate of inflation (e.g. tax on cigarettes could increase by up to 10 % above inflation).” [Tobacco policy 1]	“High strength drinks should be taxed at a higher rate than lower strength drinks.” [Alcohol policy 1] a
Availability/licensing	“Restricting the sale of cigarettes and tobacco in close proximity to schools.” [Tobacco policy 2] “Reducing the number of retailers selling cigarettes and tobacco in neighbourhoods with a high density of tobacco retailers.” [Tobacco policy 3] “Requiring anyone selling tobacco to have a licence which can be removed if they sell to those under-age.” [Tobacco policy 4]	“Public health should be considered when licence applications are made for alcohol outlets.” [Alcohol policy 2] “The sale of alcohol in shops should be restricted to between 10am and 10pm (as already implemented in Scotland).” [Alcohol policy 3] “Alcohol products should be less visible in supermarkets and shops (i.e. restrictions on how alcohol is displayed).” [Alcohol policy 4]
Industry disclosure	“Requiring the tobacco industry to publicly disclose business information relevant to its activities (such as sales data, details of lobbying and marketing).” [Tobacco policy 5]	“Require the alcohol industry to publicly disclose business information relevant to its activities (such as sales data, details of lobbying and marketing).” [Alcohol policy 5]
Treatment service	“Ensuring that every smoker who wants it can get support that is clinically proven to help stop smoking.” [Tobacco policy 6]	“Everybody who needs support for alcohol problems should be able to access it.” [Alcohol policy 6]
Health warnings	“Requiring health warnings on cigarette sticks and rolling tobacco papers.” [Tobacco policy 7]	“All products labels should feature health warnings designed by an independent health body.” [Alcohol policy 7]

a The wording in 2021 was: “Alcoholic drinks should be taxed in proportion to the amount of alcohol they contain.”

The sociodemographic covariates included gender (women or men), age (18–34 or 35+ years), social grade (more or less advantaged), education (post-16 qualifications: yes or no), and nation (England, Scotland, or Wales). For gender, participants identifying as non-binary were included when reporting characteristics of study participants, but numbers were too small for further analyses. Social grade, a measure of socioeconomic position according to the National Readership Survey's classification [30], was categorised as more advantaged (ABC1: high and intermediate managerial, administrative, or professional, supervisory, clerical, and junior managerial, administrative or professional occupations) or less advantaged (C2DE: skilled manual workers, semi and unskilled manual workers, state pensioners, casual or lowest grade workers, and unemployed with state benefits only). Education was measured depending on whether someone had a post-16 qualification (completed post-16 vocational course, A-levels or equivalent [at school until age 18], undergraduate degree or professional qualification, postgraduate degree, still studying, other) or not (no qualifications, completed General Certificate of Secondary Education/Certificate of Secondary Education/Ordinary Levels or equivalent [at school until age 16]).

Further covariates were related to tobacco and alcohol use behaviour. We classified participants as smoking tobacco if they responded “I smoke cigarettes (including hand-rolled) every day”, “I smoke cigarettes (including hand-rolled), but not every day”, or “I do not smoke cigarettes at all, but I do smoke tobacco of some kind (e.g. pipe, cigar or shisha)” to the question “Which of the following best applies to you?“. All others were classified as not smoking tobacco. Further, we classified participants as drinking at increasing and higher risk levels if they scored five or above on the alcohol use disorders identification test – consumption (AUDIT-C) score [31].

### 2.3 Analysis

We conducted a complete-cases analysis as less than 5 % of data were missing [32]. The number and proportion of missing values of each variable is reported in the supplementary material. Responses noted by the interviewer as “Don't know” or “Refused” for any question beside those about policy support were considered missing. Data from all three years were combined and analysed cross-sectionally. All data were weighted using raking [33] to match the population of Great Britain. Unweighted data are presented in the supplementary material. First, we calculated for each policy the percentage of people who stated supporting, opposing or being indecisive. Second, we stratified level of support by whether people smoked for tobacco control policies and whether they drank at increasing and higher risk levels for alcohol control policies, and further compared support among those engaging in none, one, or both behaviours. Third, we assessed the percentage of support stratified by gender, age, social grade, education, and nation.

We also added unplanned regression analyses in which we assessed potential associations between support for the different policies and behavioural or sociodemographic characteristics when adjusting for the other factors (smoking, increasing and higher risk drinking, gender, social grade, education, nation). The analysis was conducted in RStudio (version 2022.07.2, R version 4.2.1).

## 3 Results

A total of 6311 participants (unweighted) who had complete data on all relevant variables (representing 95.5 % of all participants interviewed at the time; see Supplementary Table S1 for missing values) were included in the study. Participant characteristics are presented in Table 2.

**Table 2:** Characteristics of participants (N_unweighted_ = 6311).

	Participants n (%)	
	unweighted	weighted
Age 18–34	1475 (23.4)	1813 (28.8)
Women	3160 (50.1)	3217 (51.1)
Men	3116 (49.4)	3049 (48.4)
Non-binary	35 (0.6)	35 (0.6)
England	4589 (72.7)	5444 (86.4)
Scotland	1119 (17.7)	541 (8.6)
Wales	603 (9.6)	315 (5.0)
Social grades ABC1	4261 (67.5)	3544 (56.3)
Post-16 qualification	4757 (75.4)	4711 (74.8)
Tobacco smoking	897 (14.2)	988 (15.7)
Increasing and higher risk drinking	2175 (34.5)	2137 (33.9)

### 3.1 Overall level of support for policies

**Fig. 1:**
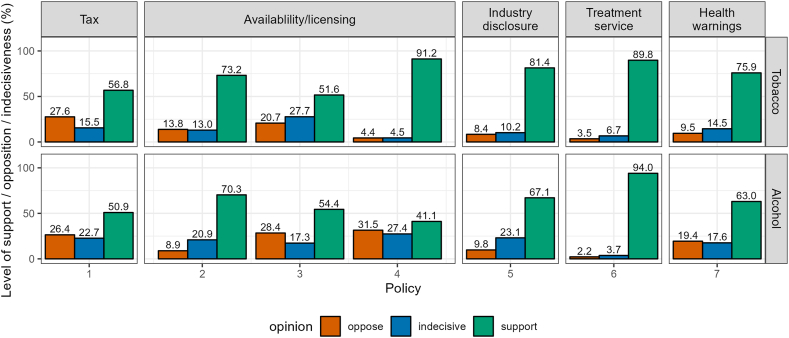
Overall level of support/opposition/indecisiveness for tobacco and alcohol control policies. See Table 1 for exact wording of each policy, indexed as Tobacco policies 1–7 and Alcohol policies 1–7.

In general, more people were in support of tobacco than alcohol control policies (Fig. 1, estimates with 95 % CIs and unweighted data in Supplementary Tables S3–4 and Fig. S1). Both tax-related policies [Tobacco policy 1 and Alcohol policy 1] received similar support, with people being slightly more indecisive about alcohol taxation. The policy to require anyone selling tobacco to have a licence which can be removed if they sell to those minors [Tobacco policy 4] was also highly supported (91.2 %). The policy that received the lowest level of support and highest level of opposition with 41.1 % and 31.5 %, respectively, was about restricting how retailers can display alcohol [Alcohol policy 4]. The policy requiring industry to publicly disclose business information relevant to its activities [Tobacco policy 5 and Alcohol policy 5] was less popular when applied to the alcohol industry (81.4 % vs. 67.1 %). This response was driven by more people feeling indecisive about the policy rather than opposing it when comparing it to the tobacco industry-related policy. Both policies about offering treatment services [Tobacco policy 6 and Alcohol policy 6] received the highest level of support (89.9 % for tobacco and 94.0 % for alcohol), with the alcohol-treatment policy receiving less opposition (6.7 % vs. 3.7 %). Between the two policies related to health warnings [Tobacco policy 7 and Alcohol policy 7], the alcohol policy received a 10 percentage points higher level of opposition and slightly more people felt indecisive about it (3 percentage points difference).

### 3.2 Support depending on smoking and drinking status

**Fig. 2:**
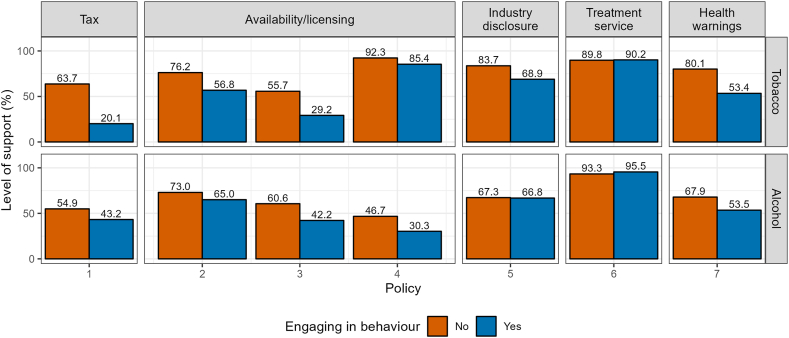
Support depending on whether people engage in behaviour – for tobacco control policies smoking tobacco and for alcohol control policies drinking at increasing and higher risk levels. See Table 1 for exact wording of each policy, indexed as Tobacco policies 1–7 and Alcohol policies 1–7.

People who engaged in the behaviour targeted by the policy were generally less supportive of it (Fig. 2, estimates with 95 % CIs and unweighted data in Supplementary Tables S5–6 and Fig. S2). This difference was more pronounced for tobacco than for alcohol. People who smoked showed lower levels of support for tobacco policies relative to those who did not smoke and vice versa for people drinking at increasing and higher risk levels (vs. not) for alcohol control policies. The biggest difference was for the policy to increase tobacco-related tax [Tobacco policy 1], with support among non-smoking people being 63.7 % compared to 20.1 % among smoking people. In comparison, the alcohol-related policy [Alcohol policy 1] received 54.9 % support among people not drinking at increasing and higher risk levels compared with 43.2 % among those who did. The policy related to reducing tobacco retailer numbers in neighbourhoods with a high density [Tobacco policy 3] received double the level of support among non-smoking adults compared with smoking adults. The policy about health warnings on cigarettes and rolling paper [Tobacco policy 7] received 80.1 % support among people not smoking compared to 53.4 % among those who did. For policies that generally received a high level of support, the difference between those engaging in the behaviour and those who did not was marginal. These descriptive results are in line with the results of the adjusted regression analyses (see Supplementary Table S10).

**Fig. 3:**
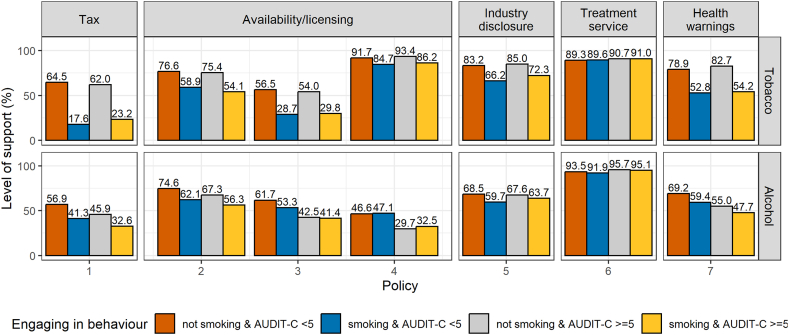
Support depending on whether people engage in none, one, or both behaviours. See Table 1 for exact wording of each policy, indexed as Tobacco policies 1–7 and Alcohol policies 1–7.

When comparing support depending on whether people engaged in none, one, or both behaviours (i.e., smoking and drinking at increasing and higher risk levels), the greatest differences were for some of the tobacco control policies, but usually the level of support was similar among those engaging only in the behaviour targeted by the policy or engaging in both behaviours (Fig. 3, estimates with 95 % CIs and unweighted data in Supplementary Tables S7–8 and Fig. S3). A noteworthy difference was for the tobacco-related tax policy [Tobacco policy 1] which found a 6 percentage points lower support among people only smoking than those engaging in both behaviours. The difference for the alcohol-related tax policy [Alcohol policy 1] was in the opposite direction: 13 percentage points lower support for those engaging in both behaviours compared with those only drinking at increasing and higher risk levels. There were no noteworthy differences in level of support for tobacco control policies between people who engaged in none of the behaviours or those who drank at increasing and higher risk levels (but did not smoke). In contrast, the level of support for alcohol control policies was usually lower among people who smoked (but did not drink at increasing and higher risk levels) than among those who engaged in none of the behaviours.

### 3.3 Level of support across different subgroups

**Fig. 4:**
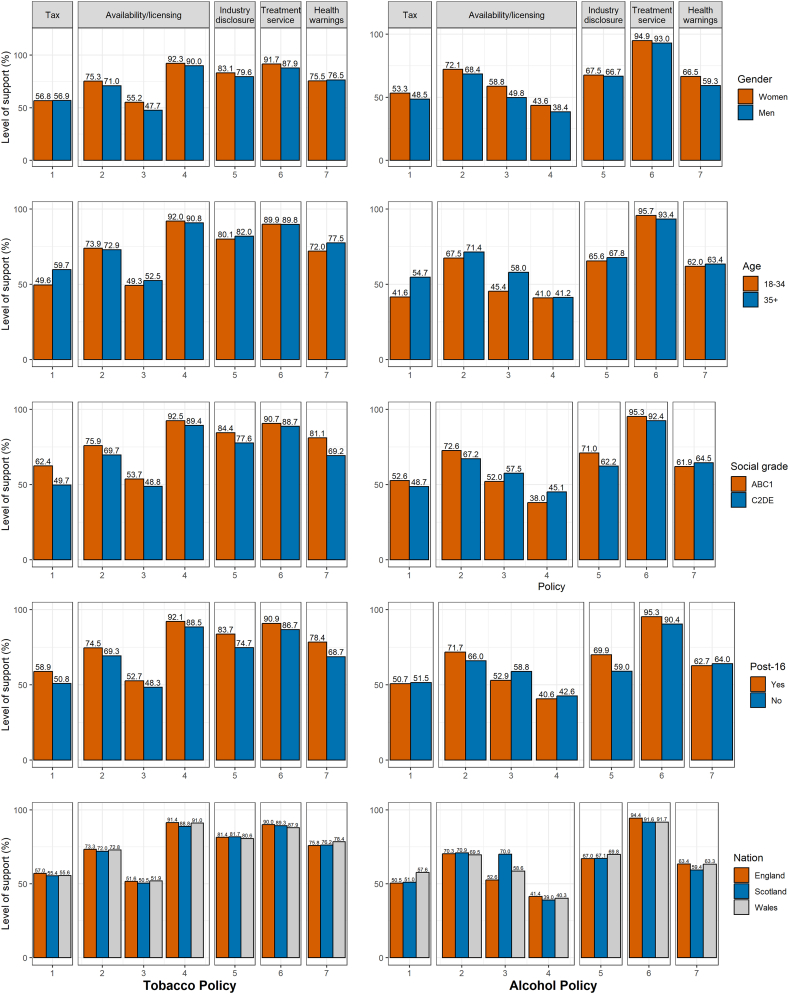
Support for tobacco (left) and alcohol (right) control policies depending on different sociodemographic characteristics. See Table 1 for exact wording of each policy, indexed as Tobacco policies 1–7 and Alcohol policies 1–7.

Generally, women and men showed fairly similar levels of support for the different policies (Fig. 4; estimates with 95 % CIs and unweighted data in Supplementary Tables S9–10 and Fig. S4). When there were differences by gender, men tended to show slightly lower levels of support. The most notable gender difference was for the policy about restrictions on alcohol sales hours in shops [Alcohol policy 3], with a difference of 9 percentage points. Similarly, in the adjusted regression analyses (Supplementary Table S10), the odds of supporting the policies were either similar between men and women, or men had slightly lower odds. Noticeable differences were apparent for the policies related to access to treatment services [Tobacco policy 6 and Alcohol policy 6], for which men had lower odds of supporting them than women.

There were some more pronounced differences between the younger (18–34 years) and the older age group (35+ years). These were for the tax-related policies [Tobacco policy 1 and Alcohol policy 1] and the policy about restricting the sales hours for alcohol in shops [Alcohol policy 3]: The tobacco tax policy received 10 percentage points less support and the other two both 13 percentage points less support from the younger group. These differences were also evident in the adjusted regression analyses (Supplementary Table S10).

10.13039/100019769People from more advantaged social grades (ABC1) showed higher levels of support for tobacco policies than those from less advantaged social grades (C2DE; Fig. 4). The biggest difference between the groups was notable in relation to tobacco taxation [Tobacco policy 1], with people from C2DE reporting 13 percentage points less support than those from ABC1. There was a similarly large difference (12 percentage points) for the policy to require health warnings on cigarette sticks and rolling papers [Tobacco policy 7]. Among alcohol policies, some received higher levels of support from those in C2DE, others from those in ABC1, and others were comparable. The most pronounced differences were for the policies about industry disclosure of business information [Alcohol policy 5] and reducing product visibility in supermarkets [Alcohol policy 4], with the former being more popular among social grades ABC1 (9 percentage points difference) and the latter among social grades C2DE (7 percentage points difference). The same tendencies were observed in the adjusted regression analyses (Supplementary Table S10).

Differences by educational level for tobacco policies were in the direction of higher support from those with post-16 qualifications (Fig. 4). The biggest difference was the policy about health warnings on cigarettes sticks and rolling paper [Tobacco policy 7], with 10 percentage points less support among people without post-16 qualification. Similarly, the policies about tax [Tobacco policy 1] and industry disclosure [Tobacco policy 5] received 8 and 9 percentage points less support from people without post-16 qualification. For alcohol policies, results were more mixed. The policy about temporal sales restrictions [Alcohol policy 3] received higher levels of support among people without post-16 qualification (7 percentage points difference), while the policy to require public health to be considered in licensing applications [Alcohol policy 2] and the policy about industry disclosure [Alcohol policy 5] received noticeably less support from this group (6 and 11 percentage points difference). These findings are in line with the results of the adjusted regression analyses (Supplementary Table S10). However, in these adjusted analyses, there were also clear differences in the support for policies to improve access to treatment services [Tobacco policy 6 and Alcohol policy 6], with people without post-16 qualifications having significantly lower odds of supporting these policies than those with post-16 qualifications when adjusting for other factors.

Across nations, there was not much variation. Two policies that stood out were related to alcohol (Fig. 4). The policy to restrict sales hours, as already implemented in Scotland [Alcohol policy 3], received by far the highest support in Scotland, followed by Wales, and then England. The tax-related policy [Alcohol policy 1] was most supported in Wales, with comparable levels in England and Scotland. These differences were also observed in the adjusted regression analyses (Supplementary Table S10), with additionally a difference in support for the alcohol policy about access to treatment services [Alcohol policy 6], with people in Scotland and Wales seemingly having lower odds of supporting the policy than people in England, when adjusting for other factors.

## 4 Discussion

In this study, we identified five patterns that others have reported regarding public health policy support. First, people who are directly affected by the policy, tend to be less likely to support it [15]. The lower prevalence of tobacco smoking (16 % among participants) compared to increasing and higher risk drinking (34 % among participants) likely contributes to lower overall support for alcohol than tobacco policies. In the study, there was less support for tobacco or alcohol policies among people smoking or drinking at increasing and higher risk levels, respectively, than among those who did not engage in the targeted behaviour. Except for the policy to improve access to treatment services. People who smoke or drink may be more supportive because they can imagine benefitting from the services in the future. Women showed overall slightly higher support for the policies than men. It may also be related at least to some extent to women in Great Britain having a lower prevalence of smoking and drinking at increasing and higher risk levels than men and would therefore be less effected by any policy changes [34,35], but also a general tendency of women being more supportive of public health policies [15]. Generally, to gain also support from people affected by the policy, it can be beneficial, when framing a policy, to attribute the health problem to society rather than the individual and emphasise the different advantages of the policy to individuals and the wider society [[36], [37], [38], [39]].

Further, groups that tend to have less disposable income (i.e., younger people, those with less advantaged social grade, and those without post-16 qualification) expressed lower support for tax policies than those with more disposable income. The age differences are also in in line with findings from the most recent British Social Attitudes report [40] which found that although people under 35 were more concerned about inequalities than older people, the younger group tended to be less supportive of taxation and social welfare spending, supporting the idea of individual social and moral choices rather than societal enforcement. Between more and less socioeconomically advantaged, the difference in support for the alcohol tax policy was less pronounced than for the tobacco tax policy. A potential reason is that tobacco smoking is more prevalent among less socioeconomically advantaged groups with the opposite being true for increasing and higher risk drinking [[41], [42], [43]]. This point may also be reflected in the study, suggesting that people who drank at increasing and higher risk levels showed higher support for the alcohol tax policy than people who smoked for the tobacco tax policy. It is noteworthy that health taxes can, in fact, be strategically implemented with the goal of reducing health inequalities by affecting people on low-income more than others or by deterring young people from taking up unhealthy behaviours [44,45]. However, the tax amount should be carefully set to minimise exacerbating financial hardship for vulnerable groups [44]. To increase public support, governments should explain how the extra revenue will be used and ensure that they follow through on these plans [46,47].

The second pattern we observed is related to differences between support for individual- or population level interventions and harm perceptions. In our study, we found that, across all subgroups, people were highly supportive of policies aiming at providing access to treatment services. These policies focus on individuals rather than populations or regulating industry. Tobacco and alcohol producers have a history of lobbying governments and the public to support interventions aimed at individuals rather than population-level approaches, referring to people's personal responsibility and making informed choices [[48], [49], [50]]. The PPI group's potential explanation for the low support for the policy to reduce visibility of alcohol products in shops also underlines this narrative. PPI members explained that such alcohol marketing restrictions might not be popular because they would affect everyone who drinks, while only a few would consume alcohol in harmful quantities (quotes provided in supplementary material).

A study by Fitzgerald et al. [51] exploring the British public's understanding of ‘alcohol problems’ also reported that policy proposals by the public mainly focussed on treatment services. As public support for policies is partly contingent on people's understanding that there is a problem that needs to be addressed [18], the public's inadequate understanding of the harmful effects of alcohol may be a core issue for lower support for alcohol compared with tobacco control policies. International treaties have helped to ostracise the tobacco industry who have lost credibility as a partner for governments in many countries, which does not apply to the alcohol industry [17,48,[52], [53], [54]]. For example, the new global alcohol action plan of the World Health Organization has limited the role of the alcohol industry compared to previous versions, but the industry is not excluded as the tobacco industry is by the Framework Convention on Tobacco Control [17,55]. In the UK, alcohol marketing is self-regulated by industry rather than the government and health warnings are not required on alcoholic drinks which adds to public perceptions of alcohol [17,56,57].

This differential treatment between tobacco and alcohol industry may also partially explain why more people supported the policy regarding the tobacco than the alcohol industry in our survey. People with less advantaged socioeconomic backgrounds (i.e., with less advantaged social grade or without post-16 qualification) showed lower support for these policies than those with more advantaged ones. Possibly, the latter group is generally more critical or aware of potential industry interference in population health. For example, a previous study found that among people who smoked those with a lower educational level had lower odds of holding tobacco industry denormalization beliefs (i.e., distrusting industry to tell truth, requiring companies to take responsibility for harm caused, believing industry spread misinformation about second-hand smoke) than those with a higher educational level [58].

The third pattern we found is about people generally being more likely to support less intrusive public health interventions, that is those that would directly affect consumption through regulation [15]. Among the least popular interventions in this study were those that restrict people's choice by regulating availability (i.e., reducing the number of tobacco retailers in areas with high density; restricting alcohol sales hours in shops; reducing visibility of alcohol products in shops). Overall, the least popular policy was to reduce alcohol product visibility in shops. In a recent Scottish Government consultation on restricting alcohol advertising and promotion, about three quarters of individuals who responded (as opposed to organisations or industry bodies) disagreed that the government should further restrict the visibility of alcohol in the retail environment [59].

The fourth pattern we identified is that there is generally high support for policies that are aimed at changing the behaviour of children or young people [15]. In our study, the policy requiring anyone selling tobacco to have a licence which can be removed if they sell to those under-age was among the policies gaining the highest levels of support. This was reflected across all subgroups. The policy restricting the sale of tobacco products near schools also received relatively high levels of support, even though not quite as high as the licensing policy, which may be related to the second being perceived as more intrusive. It would be interesting to test whether the policy reducing alcohol product visibility in shops would receive more support if the statement included an explanation that it would reduce youth exposure to the marketing of alcohol products. In comparison, tobacco displays at point-of-sale have been banned in the UK for around 10 years [60]. In a survey prior to the policy implementation, 73 % of adults in Great Britain stated that they supported the removal of tobacco displays to protect children [61].

The fifth pattern is linked to the fact that public support generally tends to increase after a public health policy has been implemented [15], which may be reflected in this study in relation to the policy about restrictions on alcohol sales hours in shops. This legislation applies in Scotland since 2008 [62], but not in England and Wales. Generally, this policy was one of the least supported, potentially because people found it too intrusive. However, the Scottish case may show that this perception could change following implementation in England and Wales. In general, it appears that public support for further regulations in a policy area grows following successful implementation, that means not just for the specific policy but more widely. According to political theory, people may be biased towards the status quo when there is uncertainty around individual gainers and losers of a reform, while after implementation support for the reform may increase because the uncertainty is resolved [63]. The UK has a long history of tobacco control regulations [52] and support for it remains high. In contrast, a comparison of six European nations showed how strictness of a country's formal alcohol control policy correlated with public attitudes towards governmental responsibility to regulate alcohol consumption with the UK scoring somewhere in the middle on both [64].

It is also important to remember that policies are shaped by political processes and once implemented, influence future policy design by shaping public and group responses [65]. Furthermore, the framing of issues and assumptions about capacities of individuals and organisations shape policy debate, affecting how target groups perceive the problem and whether they view their interests as publicly legitimate [65]. The UK Government's sustained efforts to regulate tobacco have led to significant reductions in smoking rates [3], with continued commitment to further tobacco control measures (e.g., plans for a smokefree generation policy [66]). In contrast, alcohol remains less regulated and is less frequently addressed as a public health issue.

The study findings are important as public opinion is one of the key factors considered during the policy making process [13,14]. However, other factors also play a role. According to the Advocacy Coalition Framework [67,68], external events that contribute to the emergence of new policies include changes in socioeconomic conditions, changes in public opinion, elections, and policy decisions and impacts from other subsystems (e.g., environmental protection may influence tobacco control [69]). That means that potentially competing factors impact the consideration of potential policies. For example, the tobacco industry has a history of highlighting negative social and economic impacts of policies to prevent their implementation [68,70], which could result in them not being implemented.

### 4.1 Strengths and limitations

A strength of the study is that data were collected from a representative sample of the population living in Great Britain, with only a low percentage of missing values. A limitation of the study is that to assess educational level, we classified people who reported ‘still studying’ or ‘other’ as having a post-16 qualification because the age range of study participants (18+ years) means it is more likely for them to fall under this category than not, but it is possible that we misclassified them. The sampling was not random probability but used a hybrid sampling strategy designed to minimise non-response bias, and the weighting applied aims for representativeness of the British population. Another limitation is that participants might have not heard of the proposed policies prior to the survey and were not given any further information about them. Therefore, participants may have been unable to make an informed decision about whether to support them. Further, the data were collected during the acute phase and the direct aftermaths of the COVID-19 pandemic which may have impacted opinions on policies regulating health behaviours.

## 5 Conclusions

10.13039/100019769People were generally less supportive of policies if they directly affected them, and more intrusive policies, for example, about regulating product availability, tended to gain the least support. In contrast, policies aimed at offering treatment services to individuals and protecting children received high support. Overall, this study found high public support for tobacco and alcohol control policies. With one exception, the policies included in this study were supported by over half of the participants. These results suggest that the public is in favour of government regulations to reduce smoking and drinking prevalence in Great Britain.

## CRediT authorship contribution statement

**Vera Helen Buss:** Writing – original draft, Visualization, Methodology, Formal analysis, Data curation, Conceptualization. **Lion Shahab:** Writing – review & editing, Methodology, Funding acquisition, Conceptualization. **Sharon Cox:** Writing – review & editing, Methodology, Conceptualization. **Loren Kock:** Writing – review & editing, Methodology, Conceptualization. **Melissa Oldham:** Writing – review & editing, Methodology, Conceptualization. **Linda Bauld:** Writing – review & editing, Methodology, Funding acquisition, Conceptualization. **Hazel Cheeseman:** Writing – review & editing, Methodology, Conceptualization. **Garth Reid:** Writing – review & editing, Methodology, Conceptualization. **Jamie Brown:** Writing – review & editing, Validation, Methodology, Funding acquisition, Data curation, Conceptualization.

## Ethical approval

The University College London Ethics Committee granted ethical approval for the Smoking and Alcohol Toolkit Study (ID 0498/001, Amendment request 2808/005 approved July 21, 2022). All participants provided informed verbal consent, which was approved by the ethics committee.

## Data availability

Deidentified participant data and the command syntax for the statistical analyses will be available with publication on the Open Science Framework: https://osf.io/cg2x5/.

## Funding

This work was supported by Cancer Research UK (PRCRPG-Nov21\100,002) and the UK Prevention Research Partnership (MR/S037519/1), which is funded by the British Heart Foundation, Cancer Research UK, Chief Scientist Office of the Scottish Government Health and Social Care Directorates, Engineering and Physical Sciences Research Council, Economic and Social Research Council, Health and Social Care Research and Development Division (Welsh Government), Medical Research Council, National Institute for Health Research, Natural Environment Research Council, Public Health Agency (Northern Ireland), The Health Foundation and Wellcome.

The study sponsors did not have any role in the collection, analysis, and interpretation of data; in the writing of the report; and in the decision to submit the paper for publication.

For the purpose of Open Access, the author has applied a CC BY public copyright licence to any Author Accepted Manuscript version arising from this submission.

## Declaration of competing interest

10.13039/100016170JB has received unrestricted research funding from 10.13039/100004319Pfizer and J&J, who manufacture smoking cessation medications. LK's salary is supported by the US 10.13039/100009210Food and Drug Administration and the 10.13039/100000026National Institute on Drug Abuse. LS has received honoraria for talks, unrestricted research grants and travel expenses to attend meetings and workshops from manufactures of smoking cessation medications (Pfizer; J&J) and has acted as paid reviewer for grant awarding bodies and as a paid consultant for health care companies. All authors declare no financial links with tobacco and alcoholic beverage companies, e-cigarette manufacturers, or their representatives.
