# Supplementary material for: Exploring public support for novel tobacco and alcohol control policies in Great Britain 2021–2023: A population-based cross-sectional survey
Source: Heliyon. 2024 Dec 16;11(1):e41303. doi: 10.1016/j.heliyon.2024.e41303 (PMC11728982; doi:10.1016/j.heliyon.2024.e41303)
Supplement: Multimedia component 1 [file mmc1.docx]

**Supplementary material**

**Article:** “Exploring public support for novel tobacco and alcohol control policies in Great Britain 2021-2023: A population-based cross-sectional survey”

**Contents**

[1. Missing values 1](#_Toc182997630)

[2. Overall level of support 1](#_Toc182997631)

[3. Support depending on whether people engage in behaviour 3](#_Toc182997632)

[4. Level of support across different subgroups 6](#_Toc182997633)

[5. Regression analyses 11](#_Toc182997634)

[6. Quotes from the Public and Patient Involvement group 12](#_Toc182997635)

# 1. Missing values

Table S1: Missing values for each variable included in the analysis (N_unweighted_=6609).

|  | **Missing values, n (%)** |
| --- | --- |
| All policy statement questions | 0 (0) |
| Nation | 0 (0) |
| Age | 0 (0) |
| Gender | 26 (0.4) |
| Social grade | 0 (0) |
| Education level | 66 (1.00) |
| Smoking status | 42 (0.6) |
| AUDIT-C score | 184 (2.8) |

Flow chart of number of people asked about policy support and number of people with complete data:

# 2. Overall level of support

Table S2: Weighted overall level of support for tobacco and alcohol control policies (N_unweighted_=6311).

| **Policy** | | **Estimate, % (95% CI)** | | |
| --- | --- | --- | --- | --- |
|  |  | Support | Indecisive | Oppose |
| Tobacco | 1 – Tax | 56.8 (55.4, 58.2) | 15.5 (14.5, 16.6) | 27.6 (26.3, 28.9) |
|  | 2 – Restrict retail in school proximity | 73.2 (72.0, 74.4) | 13.0 (12.1, 13.9) | 13.8 (12.8, 14.8) |
|  | 3 – Limit retail in high density area | 51.6 (50.1, 53.0) | 27.7 (26.5, 29.0) | 20.7 (19.6, 21.9) |
|  | 4 – Retail license | 91.2 (90.4, 92.0) | 4.5 (3.9, 5.0) | 4.4 (3.8, 4.9) |
|  | 5 – Industry disclosure | 81.4 (80.3, 82.5) | 10.2 (9.3, 11.0) | 8.4 (7.6, 9.2) |
|  | 6 – Treatment service | 89.8 (89.0, 90.7) | 6.7 (6.0, 7.4) | 3.5 (3.0, 4.0) |
|  | 7 – Health warnings | 75.9 (74.7, 77.2) | 14.5 (13.5, 15.5) | 9.5 (8.7, 10.4) |
| Alcohol | 1 – Tax | 50.9 (49.5, 52.3) | 22.7 (21.5, 23.9) | 26.4 (25.1, 27.6) |
|  | 2 – Retail public health consideration | 70.3 (69.0, 71.6) | 20.9 (19.7, 22.0) | 8.9 (8.1, 9.7) |
|  | 3 – Temporal sale restrictions | 54.4 (53.0, 55.8) | 17.3 (16.2, 18.3) | 28.4 (27.1, 29.6) |
|  | 4 – Reduce visibility in retail | 41.1 (39.7, 42.5) | 27.4 (26.2, 28.7) | 31.5 (30.2, 32.7) |
|  | 5 – Industry disclosure | 67.1 (65.8, 68.5) | 23.1 (21.9, 24.3) | 9.8 (8.9, 10.6) |
|  | 6 – Treatment service | 94.0 (93.4, 94.7) | 3.7 (3.2, 4.3) | 2.2 (1.8, 2.6) |
|  | 7 – Health warnings | 63.0 (61.7, 64.4) | 17.6 (16.6, 18.7) | 19.4 (18.3, 20.5) |


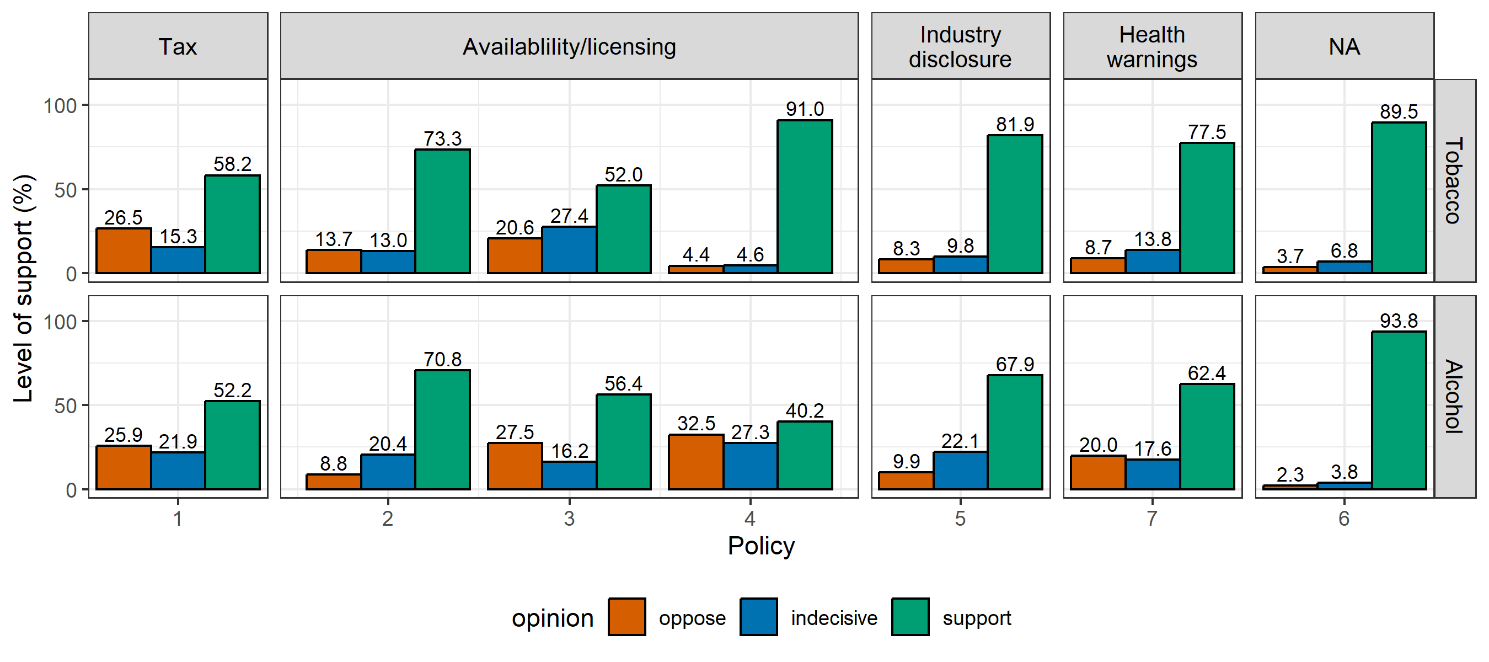


Figure S1: Overall level of support/opposition/indecisiveness (unweighted) for tobacco and alcohol control policies. See Table 1 for exact wording of each policy, indexed as Tobacco policies 1-7 and Alcohol policies 1-7.

Table S3: Unweighted overall level of support/opposition/indecisiveness for tobacco and alcohol control policies (N_unweighted_=6311).

| **Policy** | | **Estimate, %** | | |
| --- | --- | --- | --- | --- |
|  |  | Support | Indecisive | Oppose |
| Tobacco | 1 – Tax | 58.2 | 58.2 | 58.2 |
|  | 2 – Restrict retail in school proximity | 73.3 | 73.3 | 73.3 |
|  | 3 – Limit retail in high density area | 52.0 | 27.4 | 20.6 |
|  | 4 – Retail license | 91.0 | 4.6 | 4.4 |
|  | 5 – Industry disclosure | 81.9 | 9.8 | 8.3 |
|  | 6 – Treatment service | 89.5 | 6.8 | 3.7 |
|  | 7 – Health warnings | 77.5 | 13.8 | 8.7 |
| Alcohol | 1 – Tax | 52.2 | 21.9 | 25.9 |
|  | 2 – Retail public health consideration | 70.8 | 20.4 | 8.8 |
|  | 3 – Temporal sale restrictions | 56.4 | 16.2 | 27.5 |
|  | 4 – Reduce visibility in retail | 40.2 | 27.3 | 32.5 |
|  | 5 – Industry disclosure | 67.9 | 22.1 | 9.9 |
|  | 6 – Treatment service | 93.8 | 3.8 | 2.3 |
|  | 7 – Health warnings | 62.4 | 17.6 | 20.0 |

# 3. Support depending on whether people engage in behaviour

Table S4: Weighted support depending on whether people engage in behaviour – for tobacco control policies smoking tobacco and for alcohol control policies drinking at increasing and higher risk levels (N_unweighted_=6311).

| **Policy** | | **Estimate, % (95% CI)** | |
| --- | --- | --- | --- |
|  |  | No | Yes |
| Tobacco | 1 – Tax | 63.7 (62.2, 65.1) | 20.1 (17.2, 23.0) |
|  | 2 – Restrict retail in school proximity | 76.2 (75.0, 77.5) | 56.8 (53.1, 60.5) |
|  | 3 – Limit retail in high density area | 55.7 (54.2, 57.2) | 29.2 (25.7, 32.6) |
|  | 4 – Retail license | 92.3 (91.5, 93.1) | 85.4 (82.8, 87.9) |
|  | 5 – Industry disclosure | 83.7 (82.6, 84.9) | 68.9 (65.5, 72.3) |
|  | 6 – Treatment service | 89.8 (88.8, 90.7) | 90.2 (88.1, 92.3) |
|  | 7 – Health warnings | 80.1 (78.9, 81.4) | 53.4 (49.7, 57.1) |
| Alcohol | 1 – Tax | 54.9 (53.1, 56.6) | 43.2 (40.9, 45.5) |
|  | 2 – Retail public health consideration | 73.0 (71.4, 74.5) | 65.0 (62.7, 67.3) |
|  | 3 – Temporal sale restrictions | 60.6 (58.9, 62.3) | 42.2 (39.9, 44.6) |
|  | 4 – Reduce visibility in retail | 46.7 (44.9, 48.4) | 30.3 (28.1, 32.5) |
|  | 5 – Industry disclosure | 67.3 (65.7, 69.0) | 66.8 (64.6, 69.1) |
|  | 6 – Treatment service | 93.3 (92.4, 94.1) | 95.5 (94.6, 96.4) |
|  | 7 – Health warnings | 67.9 (66.3, 69.5) | 53.5 (51.1, 55.8) |


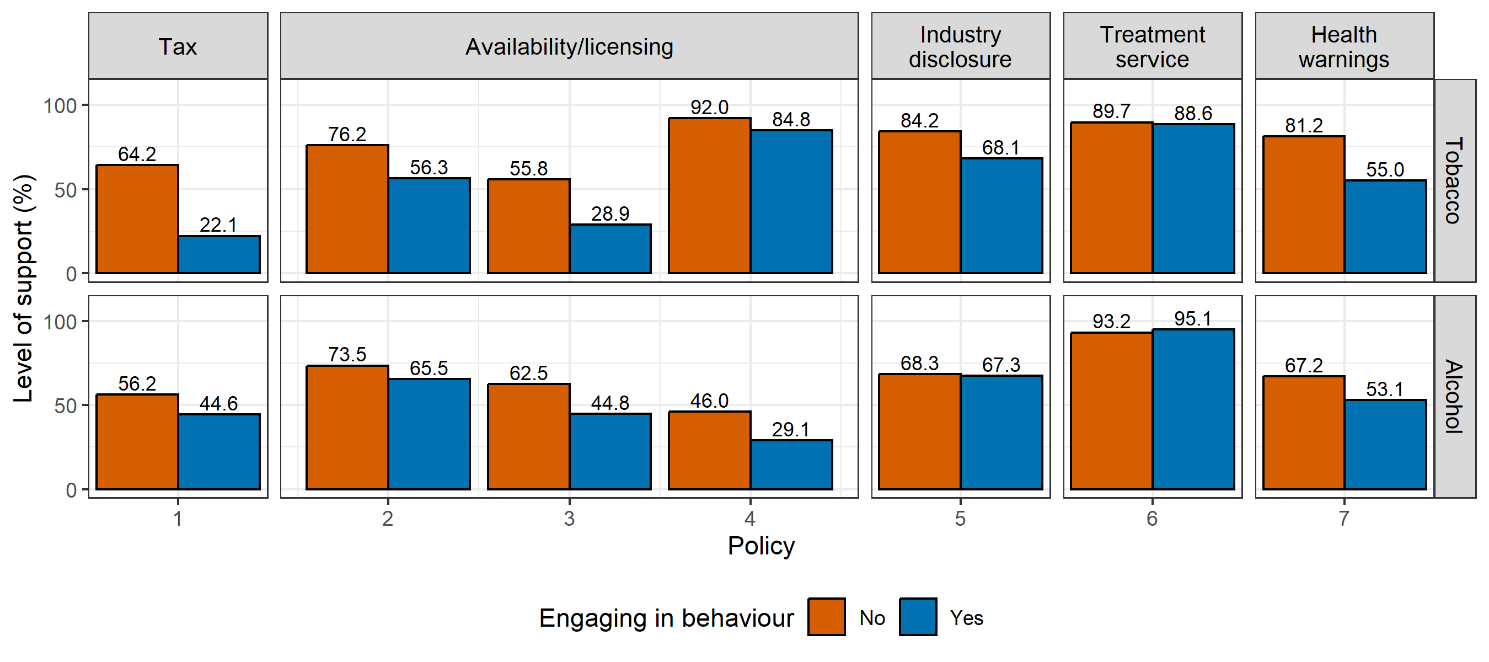


Figure S2: Support unweighted depending on whether people engage in behaviour – for tobacco control policies smoking tobacco and for alcohol control policies drinking at increasing and higher risk levels. See Table 1 for exact wording of each policy, indexed as Tobacco policies 1-7 and Alcohol policies 1-7.

Table S5: Unweighted support depending on whether people engage in behaviour – for tobacco control policies smoking tobacco and for alcohol control policies drinking at increasing and higher risk levels (N_unweighted_=6311).

| **Policy** | | **Estimate, %** | |
| --- | --- | --- | --- |
|  |  | No | Yes |
| Tobacco | 1 – Tax | 64.2 | 22.1 |
|  | 2 – Restrict retail in school proximity | 76.2 | 56.3 |
|  | 3 – Limit retail in high density area | 55.8 | 28.9 |
|  | 4 – Retail license | 92.0 | 84.8 |
|  | 5 – Industry disclosure | 84.2 | 68.1 |
|  | 6 – Treatment service | 89.7 | 88.6 |
|  | 7 – Health warnings | 81.2 | 55.0 |
| Alcohol | 1 – Tax | 56.2 | 44.6 |
|  | 2 – Retail public health consideration | 73.5 | 65.5 |
|  | 3 – Temporal sale restrictions | 62.5 | 44.8 |
|  | 4 – Reduce visibility in retail | 46.0 | 29.1 |
|  | 5 – Industry disclosure | 68.3 | 67.3 |
|  | 6 – Treatment service | 93.2 | 95.1 |
|  | 7 – Health warnings | 67.2 | 53.1 |

Table S6: Weighted support depending on whether people engage in none, one, or both behaviours (N_unweighted_=6311).

| **Policy** | | **Estimate, % (95% CI)** | |  |  |
| --- | --- | --- | --- | --- | --- |
|  |  | None | Smoking | AUDIT-C ≥5 | Both |
| Tobacco | 1 – Tax | 64.5 (62.7, 66.3) | 17.6 (13.9, 21.4) | 62.0 (59.4, 64.5) | 23.2 (18.7, 27.7) |
|  | 2 – Restrict retail in school proximity | 76.6 (75.1, 78.2) | 58.9 (54.0, 63.9) | 75.4 (73.2, 77.7) | 54.1 (48.6, 59.7) |
|  | 3 – Limit retail in high density area | 56.5 (54.7, 58.4) | 28.7 (24.0, 33.4) | 54.0 (51.3, 56.6) | 29.8 (24.8, 34.9) |
|  | 4 – Retail license | 91.7 (90.7, 92.8) | 84.7 (81.0, 88.3) | 93.4 (92.1, 94.7) | 86.2 (82.7, 89.8) |
|  | 5 – Industry disclosure | 83.2 (81.8, 84.6) | 66.2 (61.5, 71.0) | 85.0 (83.1, 86.9) | 72.3 (67.4, 77.2) |
|  | 6 – Treatment service | 89.3 (88.2, 90.5) | 89.6 (86.7, 92.4) | 90.7 (89.2, 92.2) | 91.0 (88.0, 93.9) |
|  | 7 – Health warnings | 78.9 (77.4, 80.5) | 52.8 (47.7, 57.9) | 82.7 (80.7, 84.7) | 54.2 (48.6, 59.7) |
| Alcohol | 1 – Tax | 56.9 (55.1, 58.8) | 41.3 (36.4, 46.2) | 45.9 (43.3, 48.6) | 32.6 (27.5, 37.8) |
|  | 2 – Retail public health consideration | 74.6 (73.0, 76.2) | 62.1 (57.2, 67.1) | 67.3 (64.8, 69.7) | 56.3 (50.8, 61.9) |
|  | 3 – Temporal sale restrictions | 61.7 (59.9, 63.5) | 53.3 (48.2, 58.3) | 42.5 (39.9, 45.0) | 41.4 (35.9, 46.9) |
|  | 4 – Reduce visibility in retail | 46.6 (44.8, 48.5) | 47.1 (42.0, 52.1) | 29.7 (27.3, 32.1) | 32.5 (27.3, 37.7) |
|  | 5 – Industry disclosure | 68.5 (66.7, 70.2) | 59.7 (54.7, 64.7) | 67.6 (65.1, 70.1) | 63.7 (58.4, 69.1) |
|  | 6 – Treatment service | 93.5 (92.6, 94.4) | 91.9 (89.1, 94.7) | 95.7 (94.6, 96.7) | 95.1 (93.1, 97.1) |
|  | 7 – Health warnings | 69.2 (67.5, 70.9) | 59.4 (54.4, 64.3) | 55.0 (52.4, 57.6) | 47.7 (42.1, 53.2) |


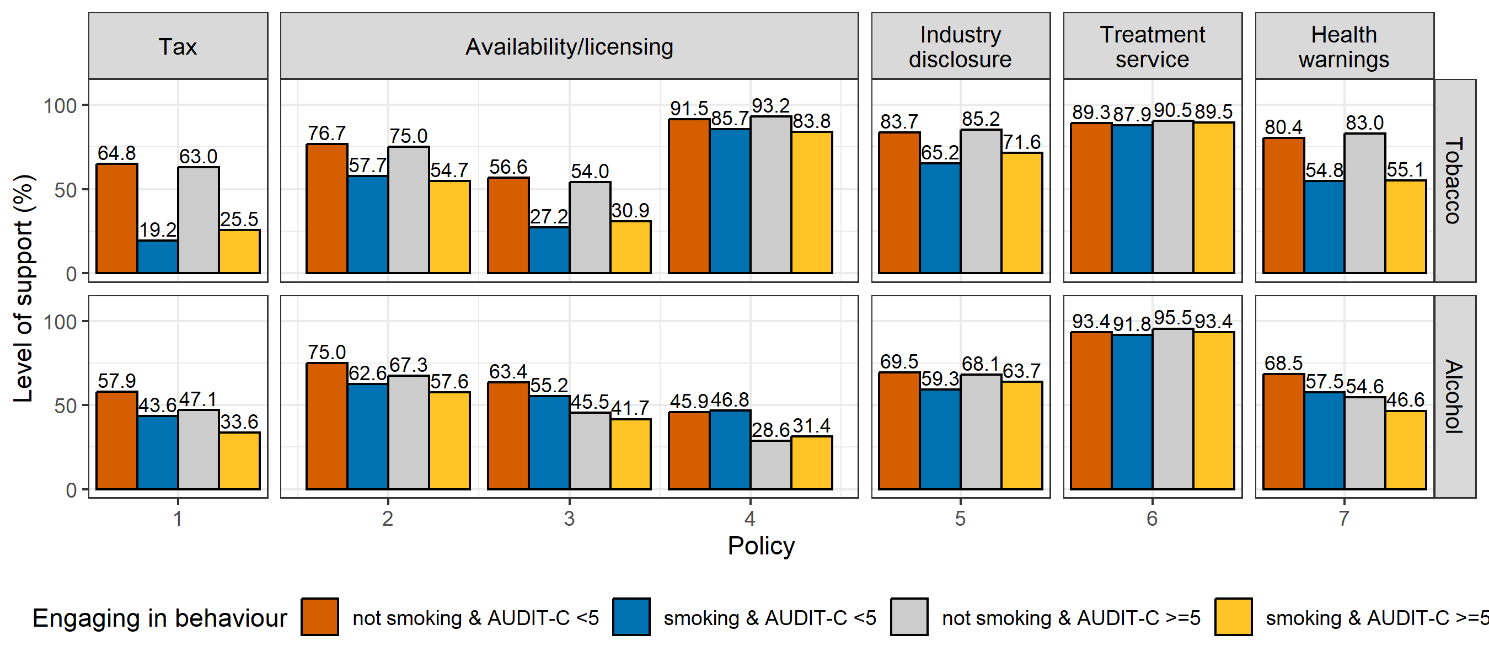


Figure S3: Support unweighted depending on whether people engage in none, one, or both behaviours. See Table 1 for exact wording of each policy, indexed as Tobacco policies 1-7 and Alcohol policies 1-7.

Table S7: Unweighted support depending on whether people engage in none, one, or both behaviours (N_unweighted_=6311).

| **Policy** | | **Estimate, %** | |  |  |
| --- | --- | --- | --- | --- | --- |
|  |  | None | Smoking | AUDIT-C ≥5 | Both |
| Tobacco | 1 – Tax | 64.8 | 19.2 | 63.0 | 25.5 |
|  | 2 – Restrict retail in school proximity | 76.7 | 57.7 | 75.0 | 54.7 |
|  | 3 – Limit retail in high density area | 56.6 | 27.2 | 54.0 | 30.9 |
|  | 4 – Retail license | 91.5 | 85.7 | 93.2 | 83.8 |
|  | 5 – Industry disclosure | 83.7 | 65.2 | 85.2 | 71.6 |
|  | 6 – Treatment service | 89.3 | 87.9 | 90.5 | 89.5 |
|  | 7 – Health warnings | 80.4 | 54.8 | 83.0 | 55.1 |
| Alcohol | 1 – Tax | 57.9 | 43.6 | 47.1 | 33.6 |
|  | 2 – Retail public health consideration | 75.0 | 62.6 | 67.3 | 57.6 |
|  | 3 – Temporal sale restrictions | 63.4 | 55.2 | 45.5 | 41.7 |
|  | 4 – Reduce visibility in retail | 45.9 | 46.8 | 28.6 | 31.4 |
|  | 5 – Industry disclosure | 69.5 | 59.3 | 68.1 | 63.7 |
|  | 6 – Treatment service | 93.4 | 91.8 | 95.5 | 93.4 |
|  | 7 – Health warnings | 68.5 | 57.5 | 54.6 | 46.6 |

# 4. Level of support across different subgroups

Table S8: Weighted support for tobacco and alcohol control policies depending on different sociodemographic characteristics (N_unweighted_=6311, for gender: N_unweighted_=6276).

| **Policy** | | **Estimate, % (95% CI)** | | | |
| --- | --- | --- | --- | --- | --- |
|  | | Women | | Men | |
| Tobacco | 1 – Tax | 56.8 (54.8, 58.8) | | 56.9 (54.9, 58.9) | |
|  | 2 – Restrict retail in school proximity | 75.3 (73.5, 77.0) | | 71.0 (69.2, 72.8) | |
|  | 3 – Limit retail in high density area | 55.2 (53.2, 57.2) | | 47.7 (45.7, 49.7) | |
|  | 4 – Retail license | 92.3 (91.3, 93.4) | | 90.0 (88.8, 91.2) | |
|  | 5 – Industry disclosure | 83.1 (81.6, 84.6) | | 79.6 (78.0, 81.3) | |
|  | 6 – Treatment service | 91.7 (90.6, 92.8) | | 87.9 (86.6, 89.2) | |
|  | 7 – Health warnings | 75.5 (73.8, 77.3) | | 76.5 (74.7, 78.2) | |
| Alcohol | 1 – Tax | 53.3 (51.3, 55.3) | | 48.5 (46.5, 50.4) | |
|  | 2 – Retail public health consideration | 72.1 (70.3, 73.9) | | 68.4 (66.5, 70.3) | |
|  | 3 – Temporal sale restrictions | 58.8 (56.8, 60.8) | | 49.8 (47.8, 51.8) | |
|  | 4 – Reduce visibility in retail | 43.6 (41.7, 45.6) | | 38.4 (36.5, 40.4) | |
|  | 5 – Industry disclosure | 67.5 (65.7, 69.4) | | 66.7 (64.8, 68.6) | |
|  | 6 – Treatment service | 94.9 (94.1, 95.8) | | 93.0 (92.1, 94.0) | |
|  | 7 – Health warnings | 66.5 (64.7, 68.4) | | 59.3 (57.3, 61.2) | |
|  | | Age 18-34 | | Age 35+ | |
| Tobacco | 1 – Tax | 49.6 (46.8, 52.5) | | 59.7 (58.2, 61.3) | |
|  | 2 – Restrict retail in school proximity | 73.9 (71.4, 76.4) | | 72.9 (71.5, 74.4) | |
|  | 3 – Limit retail in high density area | 49.3 (46.4, 52.1) | | 52.5 (50.9, 54.1) | |
|  | 4 – Retail license | 92.0 (90.5, 93.6) | | 90.8 (89.9, 91.8) | |
|  | 5 – Industry disclosure | 80.1 (77.8, 82.4) | | 82.0 (80.7, 83.2) | |
|  | 6 – Treatment service | 89.9 (88.1, 91.7) | | 89.8 (88.9, 90.8) | |
|  | 7 – Health warnings | 72.0 (69.4, 74.6) | | 77.5 (76.2, 78.9) | |
| Alcohol | 1 – Tax | 41.6 (38.8, 44.4) | | 54.7 (53.1, 56.3) | |
|  | 2 – Retail public health consideration | 67.5 (64.8, 70.1) | | 71.4 (70.0, 72.8) | |
|  | 3 – Temporal sale restrictions | 45.4 (42.6, 48.3) | | 58.0 (56.4, 59.6) | |
|  | 4 – Reduce visibility in retail | 41.0 (38.2, 43.8) | | 41.2 (39.6, 42.8) | |
|  | 5 – Industry disclosure | 65.6 (62.8, 68.3) | | 67.8 (66.3, 69.3) | |
|  | 6 – Treatment service | 95.7 (94.5, 96.8) | | 93.4 (92.6, 94.2) | |
|  | 7 – Health warnings | 62.0 (59.2, 64.7) | | 63.4 (61.9, 65.0) | |
|  | | Social grade ABC1 | | Social grade C2DE | |
| Tobacco | 1 – Tax | 62.4 (60.8, 63.9) | | 49.7 (47.3, 52.1) | |
|  | 2 – Restrict retail in school proximity | 75.9 (74.5, 77.3) | | 69.7 (67.5, 71.9) | |
|  | 3 – Limit retail in high density area | 53.7 (52.0, 55.3) | | 48.8 (46.4, 51.3) | |
|  | 4 – Retail license | 92.5 (91.7, 93.4) | | 89.4 (88.0, 90.9) | |
|  | 5 – Industry disclosure | 84.4 (83.2, 85.6) | | 77.6 (75.6, 79.6) | |
|  | 6 – Treatment service | 90.7 (89.8, 91.6) | | 88.7 (87.2, 90.3) | |
|  | 7 – Health warnings | 81.1 (79.9, 82.4) | | 69.2 (67.0, 71.5) | |
| Alcohol | 1 – Tax | 52.6 (51.0, 54.3) | | 48.7 (46.3, 51.1) | |
|  | 2 – Retail public health consideration | 72.6 (71.2, 74.1) | | 67.2 (65.0, 69.5) | |
|  | 3 – Temporal sale restrictions | 52.0 (50.3, 53.6) | | 57.5 (55.1, 59.9) | |
|  | 4 – Reduce visibility in retail | 38.0 (36.4, 39.6) | | 45.1 (42.7, 47.5) | |
|  | 5 – Industry disclosure | 71.0 (69.5, 72.5) | | 62.2 (59.8, 64.5) | |
|  | 6 – Treatment service | 95.3 (94.6, 96.0) | | 92.4 (91.2, 93.6) | |
|  | 7 – Health warnings | 61.9 (60.3, 63.4) | | 64.5 (62.2, 66.8) | |
|  | | Post-16 qualification | | No post-16 qualification | |
| Tobacco | 1 – Tax | 58.9 (57.3, 60.5) | | 50.8 (48.0, 53.7) | |
|  | 2 – Restrict retail in school proximity | 74.5 (73.1, 75.9) | | 69.3 (66.7, 72.0) | |
|  | 3 – Limit retail in high density area | 52.7 (51.0, 54.3) | | 48.3 (45.4, 51.1) | |
|  | 4 – Retail license | 92.1 (91.2, 92.9) | | 88.5 (86.7, 90.4) | |
|  | 5 – Industry disclosure | 83.7 (82.5, 84.9) | | 74.7 (72.2, 77.1) | |
|  | 6 – Treatment service | 90.9 (90.0, 91.8) | | 86.7 (84.8, 88.6) | |
|  | 7 – Health warnings | 78.4 (77.0, 79.8) | | 68.7 (65.9, 71.4) | |
| Alcohol | 1 – Tax | 50.7 (49.1, 52.3) | | 51.5 (48.7, 54.4) | |
|  | 2 – Retail public health consideration | 71.7 (70.2, 73.2) | | 66.0 (63.3, 68.7) | |
|  | 3 – Temporal sale restrictions | 52.9 (51.0, 54.5) | | 58.8 (55.9, 61.6) | |
|  | 4 – Reduce visibility in retail | 40.6 (39.0, 42.2) | | 42.6 (39.8, 45.5) | |
|  | 5 – Industry disclosure | 69.9 (68.4, 71.4) | | 59.0 (56.1, 61.8) | |
|  | 6 – Treatment service | 95.3 (94.6, 95.9) | | 90.4 (88.7, 92.1) | |
|  | 7 – Health warnings | 62.7 (61.1, 64.2) | | 64.0 (61.2, 66.7) | |
|  | | England | Scotland | | Wales |
| Tobacco | 1 – Tax | 57.0 (55.5, 58.6) | 55.4 (52.3, 58.6) | | 55.6 (51.3, 60.0) |
|  | 2 – Restrict retail in school proximity | 73.3 (71.9, 74.7) | 72.0 (69.1, 74.9) | | 72.8 (68.9, 76.7) |
|  | 3 – Limit retail in high density area | 51.6 (50.1, 53.2) | 50.5 (47.3, 53.7) | | 51.9 (47.6, 56.2) |
|  | 4 – Retail license | 91.4 (90.5, 92.3) | 88.8 (86.8, 90.9) | | 91.0 (88.4, 93.5) |
|  | 5 – Industry disclosure | 81.4 (80.2, 82.7) | 81.7 (79.2, 84.3) | | 80.6 (77.2, 84.0) |
|  | 6 – Treatment service | 90.0 (89.1, 91.0) | 89.3 (87.3, 91.3) | | 87.9 (85.1, 90.6) |
|  | 7 – Health warnings | 75.8 (74.4, 77.2) | 76.2 (73.4, 79.0) | | 78.4 (74.8, 82.0) |
| Alcohol | 1 – Tax | 50.5 (48.9, 52.1) | 51.0 (47.8, 54.2) | | 57.6 (53.3, 61.9) |
|  | 2 – Retail public health consideration | 70.3 (68.8, 71.7) | 70.9 (68.0, 73.8) | | 69.5 (65.4, 73.5) |
|  | 3 – Temporal sale restrictions | 52.6 (51.0, 54.2) | 70.0 (67.1, 72.9) | | 58.6 (54.3, 62.9) |
|  | 4 – Reduce visibility in retail | 41.4 (39.8, 42.9) | 39.0 (35.9, 42.1) | | 40.3 (36.1, 44.6) |
|  | 5 – Industry disclosure | 67.0 (65.5, 68.5) | 67.1 (64.1, 70.2) | | 69.8 (65.9, 73.7) |
|  | 6 – Treatment service | 94.4 (93.7, 95.1) | 91.6 (89.8, 93.5) | | 91.7 (89.3, 94.2) |
|  | 7 – Health warnings | 63.4 (61.8, 64.9) | 59.4 (56.3, 62.5) | | 63.3 (59.1, 67.5) |


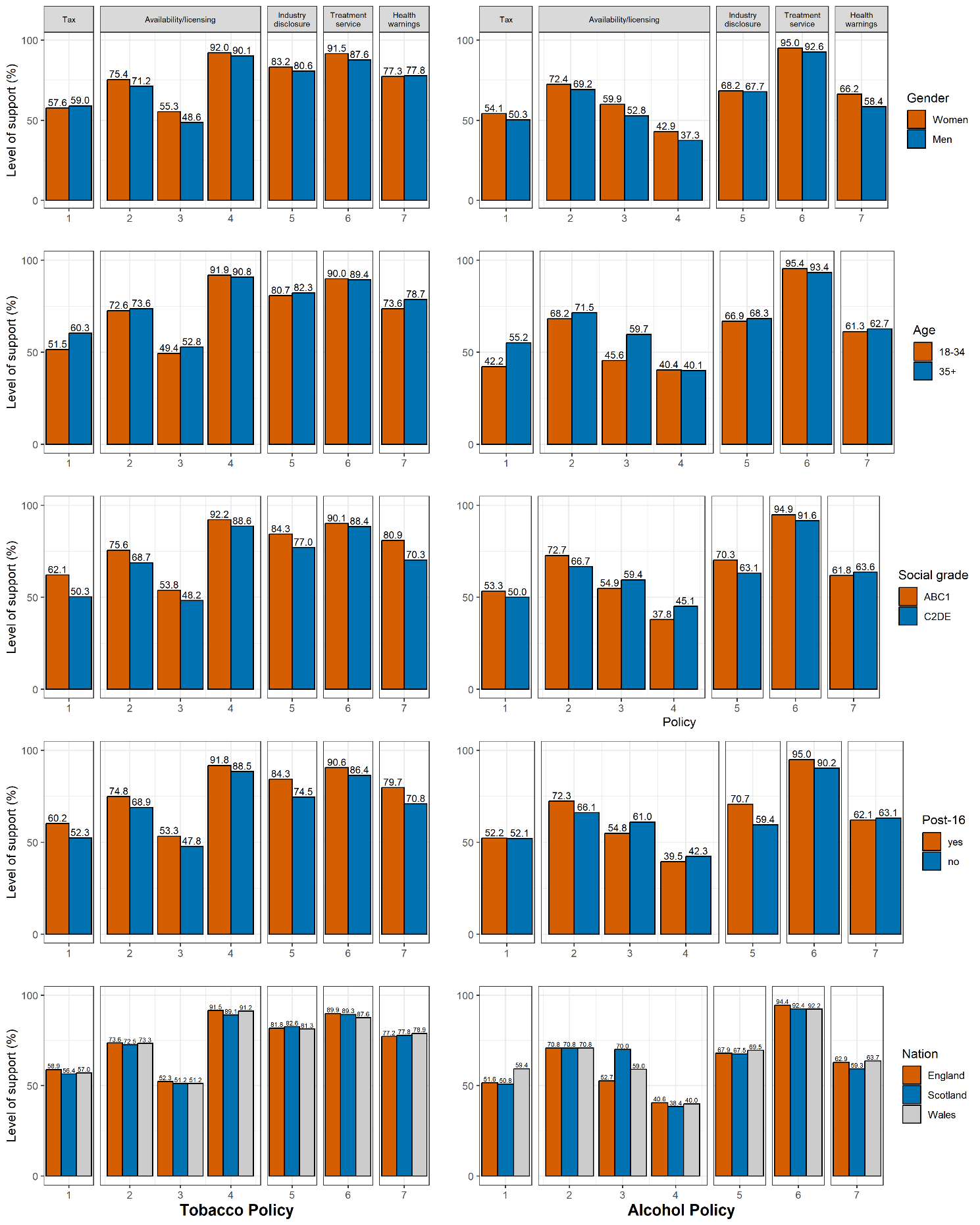


Figure S4: Support unweighted for tobacco (left) and alcohol (right) control policies depending on different sociodemographic characteristics. See Table 1 for exact wording of each policy, indexed as Tobacco policies 1-7 and Alcohol policies 1-7.

Table S9: Unweighted support for tobacco and alcohol control policies depending on different sociodemographic characteristics (N_unweighted_=6311, for gender: N_unweighted_=6276).

| **Policy** | | **Estimate, % (95% CI)** | | | |
| --- | --- | --- | --- | --- | --- |
|  | | Women | | Men | |
| Tobacco | 1 – Tax | 57.6 | | 59.0 | |
|  | 2 – Restrict retail in school proximity | 75.4 | | 71.2 | |
|  | 3 – Limit retail in high density area | 55.3 | | 48.6 | |
|  | 4 – Retail license | 92.0 | | 90.1 | |
|  | 5 – Industry disclosure | 83.2 | | 80.6 | |
|  | 6 – Treatment service | 91.5 | | 87.6 | |
|  | 7 – Health warnings | 77.3 | | 77.8 | |
| Alcohol | 1 – Tax | 54.1 | | 50.3 | |
|  | 2 – Retail public health consideration | 72.4 | | 69.2 | |
|  | 3 – Temporal sale restrictions | 59.9 | | 52.8 | |
|  | 4 – Reduce visibility in retail | 42.9 | | 37.3 | |
|  | 5 – Industry disclosure | 68.2 | | 67.7 | |
|  | 6 – Treatment service | 95.0 | | 92.6 | |
|  | 7 – Health warnings | 66.2 | | 58.4 | |
|  | | Age 18-34 | | Age 35+ | |
| Tobacco | 1 – Tax | 51.5 | | 60.3 | |
|  | 2 – Restrict retail in school proximity | 72.6 | | 73.6 | |
|  | 3 – Limit retail in high density area | 49.4 | | 52.8 | |
|  | 4 – Retail license | 91.9 | | 90.8 | |
|  | 5 – Industry disclosure | 80.7 | | 82.3 | |
|  | 6 – Treatment service | 90.0 | | 89.4 | |
|  | 7 – Health warnings | 73.6 | | 78.7 | |
| Alcohol | 1 – Tax | 42.2 | | 55.2 | |
|  | 2 – Retail public health consideration | 68.2 | | 71.5 | |
|  | 3 – Temporal sale restrictions | 45.6 | | 59.7 | |
|  | 4 – Reduce visibility in retail | 40.4 | | 40.1 | |
|  | 5 – Industry disclosure | 66.9 | | 68.3 | |
|  | 6 – Treatment service | 95.4 | | 93.4 | |
|  | 7 – Health warnings | 61.3 | | 62.7 | |
|  | | Social grade ABC1 | | Social grade C2DE | |
| Tobacco | 1 – Tax | 62.1 | | 50.3 | |
|  | 2 – Restrict retail in school proximity | 75.6 | | 68.7 | |
|  | 3 – Limit retail in high density area | 53.8 | | 48.2 | |
|  | 4 – Retail license | 92.2 | | 88.6 | |
|  | 5 – Industry disclosure | 84.3 | | 77.0 | |
|  | 6 – Treatment service | 90.1 | | 88.4 | |
|  | 7 – Health warnings | 80.9 | | 70.3 | |
| Alcohol | 1 – Tax | 53.3 | | 50.0 | |
|  | 2 – Retail public health consideration | 72.7 | | 66.7 | |
|  | 3 – Temporal sale restrictions | 54.9 | | 59.4 | |
|  | 4 – Reduce visibility in retail | 37.8 | | 45.1 | |
|  | 5 – Industry disclosure | 70.3 | | 63.1 | |
|  | 6 – Treatment service | 94.9 | | 91.6 | |
|  | 7 – Health warnings | 61.8 | | 63.6 | |
|  | | Post-16 qualification | | No post-16 qualification | |
| Tobacco | 1 – Tax | 60.2 | | 52.3 | |
|  | 2 – Restrict retail in school proximity | 74.8 | | 68.9 | |
|  | 3 – Limit retail in high density area | 53.3 | | 47.8 | |
|  | 4 – Retail license | 91.8 | | 88.5 | |
|  | 5 – Industry disclosure | 84.3 | | 74.5 | |
|  | 6 – Treatment service | 90.6 | | 86.4 | |
|  | 7 – Health warnings | 79.7 | | 70.8 | |
| Alcohol | 1 – Tax | 52.2 | | 52.1 | |
|  | 2 – Retail public health consideration | 72.3 | | 66.1 | |
|  | 3 – Temporal sale restrictions | 54.8 | | 61.0 | |
|  | 4 – Reduce visibility in retail | 39.5 | | 42.3 | |
|  | 5 – Industry disclosure | 70.7 | | 59.4 | |
|  | 6 – Treatment service | 95.0 | | 90.2 | |
|  | 7 – Health warnings | 62.1 | | 63.1 | |
|  | | England | Scotland | | Wales |
| Tobacco | 1 – Tax | 58.9 | 56.4 | | 57 |
|  | 2 – Restrict retail in school proximity | 73.6 | 72.5 | | 73.3 |
|  | 3 – Limit retail in high density area | 52.3 | 51.2 | | 51.2 |
|  | 4 – Retail license | 91.5 | 89.1 | | 91.2 |
|  | 5 – Industry disclosure | 81.8 | 82.6 | | 81.3 |
|  | 6 – Treatment service | 89.9 | 89.3 | | 87.6 |
|  | 7 – Health warnings | 77.2 | 77.8 | | 78.9 |
| Alcohol | 1 – Tax | 51.6 | 50.8 | | 59.4 |
|  | 2 – Retail public health consideration | 70.8 | 70.8 | | 70.8 |
|  | 3 – Temporal sale restrictions | 52.7 | 70.0 | | 59.0 |
|  | 4 – Reduce visibility in retail | 40.6 | 38.4 | | 40.0 |
|  | 5 – Industry disclosure | 67.9 | 67.5 | | 69.5 |
|  | 6 – Treatment service | 94.4 | 92.4 | | 92.2 |
|  | 7 – Health warnings | 62.9 | 59.3 | | 63.7 |

# 5. Regression analyses

Table S10: Adjusted odds ratios for policy support (N_unweighted_=6311). Other factors were used for adjustment.

| **Factor** | **Ref.** | **Tobacco policy support, adjusted odds ratio (95% CI)** | | | | | | |
| --- | --- | --- | --- | --- | --- | --- | --- | --- |
|  |  | 1 – Tax | 2 – Restrict retail in school proximity | 3 – Limit retail in high density area | 4 – Retail license | 5 – Industry disclosure | 6 – Treatment service | 7 – Health warnings |
| Smoking | No | 0.16 (0.13, 0.19) | 0.42 (0.36, 0.50) | 0.34 (0.28, 0.41) | 0.50 (0.39, 0.63) | 0.46 (0.38, 0.55) | 1.12 (0.85, 1.46) | 0.31 (0.26, 0.37) |
| AUDIT-C ≥5 | No | 0.89 (0.78, 1.02) | 0.90 (0.78, 1.03) | 0.96 (0.85, 1.09) | 1.23 (0.99, 1.53) | 1.17 (1.00, 1.38) | 1.24 (1.01, 1.52) | 1.13 (0.97, 1.31) |
| Gender | Women | 1.10 (0.97, 1.25) | 0.84 (0.74, 0.96) | 0.76 (0.68, 0.86) | 0.75 (0.61, 0.91) | 0.80 (0.69, 0.93) | 0.64 (0.52, 0.77) | 1.10 (0.95, 1.27) |
| Age | 18-34 | 1.35 (1.16, 1.56) | 0.87 (0.74, 1.02) | 1.03 (0.89, 1.18) | 0.84 (0.65, 1.09) | 1.13 (0.94, 1.35) | 1.10 (0.87, 1.38) | 1.26 (1.07, 1.48) |
| Social grade | ABC1 | 0.71 (0.62, 0.80) | 0.81 (0.70, 0.93) | 0.93 (0.82, 1.05) | 0.79 (0.64, 0.97) | 0.78 (0.66, 0.91) | 0.90 (0.73, 1.10) | 0.63 (0.55, 0.73) |
| Post-16 | Yes | 0.77 (0.66, 0.89) | 0.85 (0.73, 1.00) | 0.88 (0.76, 1.01) | 0.76 (0.61, 0.96) | 0.62 (0.53, 0.74) | 0.67 (0.54, 0.84) | 0.68 (0.58, 0.80) |
| Scotland | England | 0.92 (0.79, 1.08) | 0.96 (0.81, 1.13) | 0.97 (0.84, 1.12) | 0.75 (0.59, 0.96) | 1.00 (0.82, 1.21) | 0.92 (0.72, 1.17) | 1.02 (0.85, 1.22) |
| Wales | England | 0.88 (0.72, 1.07) | 0.97 (0.79, 1.20) | 0.99 (0.82, 1.20) | 0.97 (0.70, 1.36) | 0.94 (0.74, 1.20) | 0.83 (0.63, 1.10) | 1.15 (0.91, 1.46) |
| **Factor** | **Ref.** | **Alcohol policy support, adjusted odds ratio (95% CI)** | | | | | | |
|  |  | 1 – Tax | 2 – Retail public health consideration | 3 – Temporal sale restrictions | 4 – Reduce visibility in retail | 5 – Industry disclosure | 6 – Treatment service | 7 – Health warnings |
| Smoking | No | 0.60 (0.51, 0.71) | 0.63 (0.53, 0.75) | 0.82 (0.69, 0.97) | 1.02 (0.86, 1.21) | 0.82 (0.69, 0.98) | 0.89 (0.64, 1.23) | 0.68 (0.58, 0.81) |
| AUDIT-C ≥5 | No | 0.67 (0.59, 0.75) | 0.70 (0.62, 0.80) | 0.52 (0.46, 0.59) | 0.52 (0.45, 0.59) | 0.96 (0.84, 1.09) | 1.56 (1.20, 2.04) | 0.59 (0.52, 0.67) |
| Gender | Men | 0.90 (0.80, 1.01) | 0.91 (0.80, 1.03) | 0.78 (0.69, 0.88) | 0.90 (0.80, 1.01) | 0.99 (0.87, 1.12) | 0.66 (0.52, 0.85) | 0.81 (0.72, 0.91) |
| Age | 18-34 | 1.57 (1.36, 1.80) | 1.16 (1.00, 1.35) | 1.50 (1.30, 1.72) | 0.98 (0.85, 1.13) | 1.14 (0.99, 1.33) | 0.75 (0.55, 1.04) | 1.00 (0.87, 1.15) |
| Social grade | ABC1 | 0.87 (0.77, 0.99) | 0.83 (0.72, 0.95) | 1.21 (0.93, 1.25) | 1.29 (1.14, 1.47) | 0.75 (0.66, 0.86) | 0.74 (0.58, 0.94) | 1.11 (0.98, 1.27) |
| Post-16 | Yes | 0.98 (0.85, 1.13) | 0.77 (0.66, 0.89) | 1.08 (0.93, 1.25) | 0.95 (0.83, 1.10) | 0.66 (0.57, 0.76) | 0.55 (0.43, 0.72) | 0.99 (0.85, 1.14) |
| Scotland | England | 0.98 (0.85, 1.14) | 1.02 (0.87, 1.20) | 2.14 (1.82, 2.50) | 0.93 (0.80, 1.07) | 1.00 (0.86, 1.17) | 0.67 (0.51, 0.89) | 0.86 (0.74, 1.00) |
| Wales | England | 1.25 (1.03, 1.50) | 0.95 (0.77, 1.17) | 1.19 (0.98, 1.45) | 0.94 (0.77, 1.14) | 1.12 (0.91, 1.37) | 0.71 (0.49, 1.01) | 0.99 (0.81, 1.20) |

Abbreviations: CI, confidence interval; Ref., reference group.

# 6. Quotes from the Public and Patient Involvement group

Quote 1: "*Alcohol is already expensive and so many of the good deals are now gone, if you start reducing where we can see it or hide it away it means we cannot see any offers. I know this may be because you want to protect the public but not everyone drinks to extreme levels, and it is just ruining it.*"

Quote 2: "*Regarding visibility, it makes it seem like alcohol is a contraband substance, and it's not. I get why we do it for cigarettes and the like but there is not the same harm [for alcohol] unless you drink to excess, which I don't think most people do.*"

Quote 3: "*I often pick alcohol on the shape and decor of the bottle, like wine or trendy beer, I am not a wine connoisseur so if you started hiding it away, I would not know what I was buying, I wouldn't buy less. I get why it's not supported.*"
